# A Multisite Network Assessment of the Epidemiology and Etiology of Acquired Diarrhea among U.S. Military and Western Travelers (Global Travelers’ Diarrhea Study): A Principal Role of *Norovirus* among Travelers with Gastrointestinal Illness

**DOI:** 10.4269/ajtmh.20-0053

**Published:** 2020-09-21

**Authors:** Hayley R. Ashbaugh, June M. Early, Myles E. Johnson, Mark P. Simons, Paul C. F. Graf, Mark S. Riddle, Brett E. Swierczewski, for the GTD Study Team

**Affiliations:** 1 Public Health Directorate, Armed Forces Health Surveillance Division, Global Emerging Infections Surveillance, Silver Spring, Maryland;; 2 General Dynamics Information Technology, Silver Spring, Maryland;; 3 Naval Medical Research Center, Silver Spring, Maryland;; 4 Naval Health Research Center, San Diego, California;; 5 University of Nevada, Reno, Nevada;; 6 Walter Reed Army Institute of Research, Silver Spring, Maryland

## Abstract

U.S. military personnel must be ready to deploy to locations worldwide, including environments with heightened risk of infectious disease. Diarrheal illnesses continue to be among the most significant infectious disease threats to operational capability. To better prevent, detect, and respond to these threats and improve synchronization across the Department of Defense (DoD) overseas laboratory network, a multisite Global Travelers’ Diarrhea protocol was implemented with standardized case definitions and harmonized laboratory methods to identify enteric pathogens. Harmonized laboratory procedures for detection of *Norovirus* (*NoV*), enterotoxigenic *Escherichia coli* (ETEC), enteroaggregative *E. coli*, Shiga toxin–producing *E. coli*, enteropathogenic *E. coli*, *Salmonella enterica*, *Shigella*/enteroinvasive *E. coli*, and *Campylobacter jejuni* have been implemented at six DoD laboratories with surveillance sites in Egypt, Honduras, Peru, Nepal, Thailand, and Kenya. Samples from individuals traveling from wealthy to poorer countries were collected between June 2012 and May 2018, and of samples with all variables of interest available (*n* = 410), most participants enrolled were students (46%), tourists (26%), U.S. military personnel (13%), or other unspecified travelers (11%). One or more pathogens were detected in 59% of samples tested. Of samples tested, the most commonly detected pathogens were *NoV* (24%), ETEC (16%), and *C. jejuni* (14%), suggesting that *NoV* plays a larger role in travelers’ diarrhea than has previously been described. Harmonized data collection and methods will ensure identification and characterization of enteric pathogens are consistent across the DoD laboratory network, ultimately resulting in more comparable data for global assessments, preventive measures, and treatment recommendations.

## INTRODUCTION

Travelers’ diarrhea (TD) has been described as the most common medical ailment among those traveling from resource-wealthy to resource-poor countries. According to data from the Foodborne Diseases Active Surveillance Network, the highest burden of infectious diarrhea was reported among U.S. citizens returning from travel to Mexico (32.7%), India (8.2%), and Peru (4.0%).^1^ Although modern advances in public health, such as improved water, sanitation and hygiene conditions; development and widespread dissemination of vaccines; and antimicrobials to treat infection have all led to an overall decline in infectious diarrhea during U.S. military engagements, it still remains a significant threat to travelers, both civilian and military,^2,3^ even those whose travel is long term (1 month or more).^4^ Military personnel experience TD in austere, operational settings that are unique among international travelers^2,5^ and present diagnostic challenges.^6^ The 2019 U.S. Military Infectious Diseases Threats Prioritization Panel,^7^ which ranks infectious disease threats by tiers of military concern to guide medical research investment, ranked bacterial diarrhea first among 65 threats.^7^ Diarrheal illnesses continue to threaten operational capability through mission degradation and lost person-hours,^8^ with deployed military service members traveling from higher to lower income countries experiencing an approximately 30% incidence of diarrhea,^5^ and most cases of untreated TD lasting 4–5 days.^9^ There is a dire need for improved surveillance that will better define this infectious disease threat and leading to more effective prevention and treatment practices.

The Armed Forces Health Surveillance Division, Global Emerging Infections Surveillance section facilitates global surveillance of enteric pathogens across the Geographic Combatant Commands to provide data that inform force health protection (FHP) decision-making, Department of Defense (DoD) policy, public health action to prevent, detect, and respond to enteric threats, as well as research involving product development (e.g., pharmaceuticals, vaccines, and diagnostics), ultimately benefiting DoD beneficiaries worldwide. Although enteric surveillance throughout the DoD overseas laboratory network is robust, it has been hampered by a lack of integrated case definitions, standardized data elements, and nonuniversally optimized laboratory procedures. Such limitations are challenges to understanding the true burden of disease across regions. In an effort to improve harmonization and yield more comparable data, DoD partners designed and implemented a multisite Global TD (GTD) protocol consisting of standardized case definitions for enteric disease and harmonized laboratory methods for identification of enteric pathogens.

## MATERIALS AND METHODS

Our study used standardized case definitions for TD (Table 1) to include both acute diarrhea (AD) and acute gastroenteritis (AGE), a minimum set of clinical data elements and harmonized laboratory procedures for detection of *Norovirus* (*NoV*), including genogroup identification; diarrheagenic *Escherichia coli* (DEC), including toxins and colonization factors (CFs); *Salmonella enterica*; *Shigella* spp.; and *Campylobacter jejuni*. The GTD study also included a robust laboratory quality assurance and quality control (QA/QC) program and a centralized data management system.

**Table 1:** AD/AGE case definitions

AD	≥ 3 Loose/liquid stools (grades 3–5*) in the preceding 24 hours or ≥ 2 loose/liquid stools in the preceding 24 hours plus at least ≥ 2 associated gastrointestinal symptoms, including subjective fever/chills, nausea, vomiting, abdominal cramping, abdominal pain, tenesmus, bloating, fecal urgency, or gross blood in stool
AGE	≥ 3 Vomiting episodes in the preceding 24 hours with ≥ 1 additional GI symptoms (e.g., diarrhea, nausea, abdominal cramping, abdominal pain, tenesmus, bloating, or fecal urgency) or ≥ 2 vomiting episode in the preceding 24 hours with ≥ 2 additional GI symptoms (e.g., diarrhea, nausea, abdominal cramping, abdominal pain, tenesmus, bloating, or fecal urgency)

AD = acute diarrhea; AGE = acute gastroenteritis; GI = gastrointestinal.

* Stool grade: grade 1 = fully formed (normal); grade 2 = soft (normal); grade 3 = thick liquid taking form of container (unformed); grade 4 = opaque watery (unformed); grade 5 = rice water (unformed).

### Study population.

Although the GTD study incorporates eight partner laboratories in the DoD network, our analysis included six laboratories (Table 2) from the period of June 2012 to May 2018. The DoD laboratories participating in this study represent surveillance sites in Egypt, Honduras, Peru, Nepal, Thailand, and Kenya (GTD study sites in Cambodia and Georgia were not included because of few samples available for analysis). Participants were enrolled from embassy clinics, traveler clinics, foreign language schools, and military installations when they sought care for AD or AGE.

**Table 2:** Partner laboratories and participating sites of the Global Travelers’ Diarrhea study

Country	Participating laboratory	Field site(s)
Egypt	NAMRU-3	American University Clinic, E.U. Clinic, U.S. Embassy
Honduras	NAMRU-6	Joint Task Force Bravo, Soto Cano Air Base
Kenya	USAMRD-K	British Army Training Unit Kenya, U.S. Embassy
Nepal	AFRIMS	CIWEC Clinic, Kathmandu, CIWEC Clinic, Pokhara
Peru	NAMRU-6	Amauta Spanish Language School, U.S. Embassy
Thailand	AFRIMS	Travel Medicine Clinic, Phuket

AFRIMS = Armed Forces Research Institute of Medical Sciences, Bangkok, Thailand; NAMRU-3 = Naval Medical Research Unit-3, Cairo, Egypt; NAMRU-6 = Naval Medical Research Unit-6, Lima, Peru; USAMRD-K = U.S. Army Medical Research Directorate-Kenya, Nairobi, Kenya; CIWEC = Canadian International Water and Energy Consultants.

### Study eligibility.

Because previous work has shown that travelers from wealthier countries have higher attack rates than those from less wealthy countries,^10^ participants were required to originate from Organisation for Economic Co-operation and Development (OECD)^11^ member countries, with travel to OECD nonmember countries. Participants included in the study were 18 years or older and had been in the country 1 year or less. Those with reported consumption (dose and duration) of any antimicrobial agent(s) within the preceding 7 days before study enrollment date (with the exception of antimalarial agents, such as Malarone [atovaquone/proguanil combination], doxycycline, chloroquine, mefloquine, or primaquine); those with chronic, persistent gastrointestinal (GI) symptom(s) with a duration greater than 7 days before enrollment, or noninfectious diarrhea; and those who could not produce a stool sample were excluded from the study. Participants were eligible to be enrolled multiple times in the study; however, a different subject identifier was used for each new episode of AD or AGE.

After assessing eligibility and obtaining informed consent, participants underwent a clinical evaluation, provided a stool specimen, and completed a questionnaire administered by a healthcare worker. The questionnaire elicited demographic information (sex, age, country of residence, and type of travel), clinical presentation (vital signs, clinical signs and symptoms, and stool grade), treatment history and on-site treatment administered (treatment setting, treatment type, treatment provided, etc.), and case disposition (effect of illness on ability to travel or perform duties). Each site was independently responsible for developing a questionnaire to collect these harmonized predetermined minimum data elements, although questionnaire verbiage and formatting itself were not harmonized to leverage existing data collection infrastructure at the individual site level. Participants were treated for their illnesses as per site clinical treatment guidelines.

### Laboratory methods.

**Figure 1.**
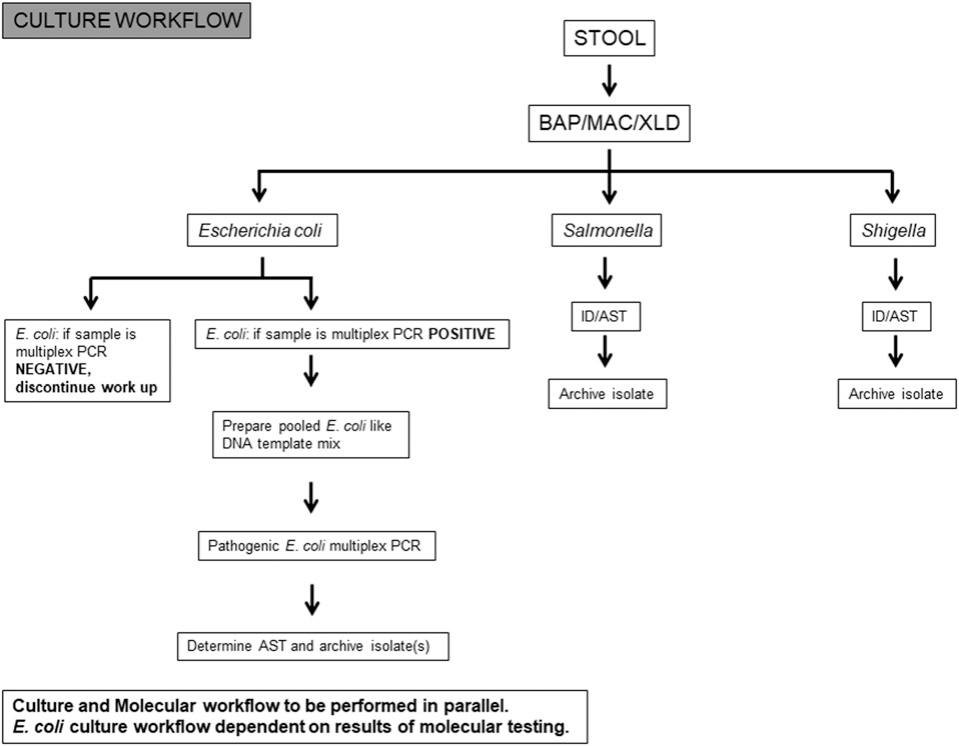
Global travelers’ diarrhea study standardized culture testing scheme. Some laboratories may have used other agars for isolation of *Salmonella*, *Shigella*, and other enteropathogens. In addition, a *Campylobacter*-selective agar plate was used (not shown). BAP = 5% sheep blood agar plate; MAC = MacConkey agar; XLD = xylose lysine deoxycholate agar.

**Figure 2.**
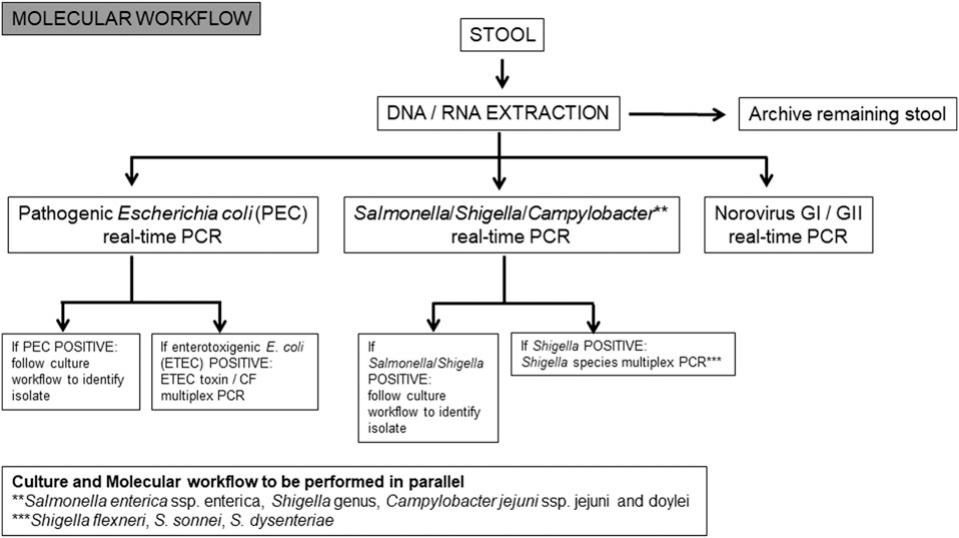
Global travelers’ diarrhea study standardized molecular testing scheme. CF = colonization factor.

Each participating laboratory tested clinical specimens in compliance with standard operating procedures (SOPs) developed by the Naval Health Research Center (NHRC) for molecular testing of the GTD study core pathogens: *NoV*, enterotoxigenic *E. coli* (ETEC), CF antigens of ETEC (ETEC-CF), enteroaggregative *E. coli* (EAEC), *Shigella*/enteroinvasive *E. coli* (EIEC), Shiga toxin–producing *E. coli* (STEC), enteropathogenic *E. coli* (EPEC), *Salmonella*, and *C. jejuni*. Three categories of testing were performed at each laboratory: 1) traditional plate-based culture, identification and antimicrobial susceptibility testing (AST), 2) bacterial isolate DNA–targeted PCR, and 3) stool RNA/DNA–targeted real-time PCR and conventional PCR. Traditional culture- (Figure 1) and molecular-based assays (Figure 2) were performed in parallel to increase the chances of identifying pathogens. For culture-based testing, stool samples were streaked onto various selective and nonselective agar plates and incubated as per the protocol. *Salmonella*, *Shigella*, and *Campylobacter* were the primary pathogens of interest, but individual laboratories may have used protocols to detect *Vibrio*, *Yersinia*, *Aeromonas*, *Plesiomonas*, and DEC as determined by each individual laboratory. Bacteria were identified by a combination of conventional microbiological methods, manual multiplex biochemical test strips, and automated identification systems, with serological confirmation performed by some laboratories. Antimicrobial susceptibility testing was performed by agar disk diffusion, gradient strips, or automated systems.

Molecular testing SOPs prepared by researchers at the NHRC were distributed to participating sites before study initiation. In brief, viral RNA was extracted via the QIAGEN (Germantown, MD) QIAamp^®^ Viral RNA Mini Kit. Testing for *NoV* GI and genogroup II (GII) RNA in fecal samples was completed using the *NoV* duplex real-time (TaqMan^®^) reverse transcriptase (RT)-PCR assay developed by the CDC as part of CaliciNet.^12^ Because findings of *NoV* infections with both GI and GII are uncommon, we have considered such findings to be a single infection for analysis purposes. A real-time multiplex PCR assay was used for the identification of *Salmonella*, *Shigella*–EIEC, and *C. jejuni* in fecal samples. Identification of DEC in extracted stool samples was performed using a multiplex assay set containing targets for EPEC, STEC, ETEC, EAEC, and EIEC. Reactions were run on an Applied Biosystems 7500 Fast Real-Time PCR System (ThermoFisher Scientific, Waltham, MA).

Detection of ETEC toxins and CFs was performed using a conventional, four-part, multiplex PCR assay. This assay was used to determine whether a lactose-fermenting, *E. coli*–like bacterial colony was ETEC and to categorize the strain based on its toxin and CF profiles. In addition, a conventional multiplex PCR assay was used for the identification of select *Shigella* species (*S. flexneri*, *S. sonnei*, and *S. dysenteriae*) in stool samples known to be *Shigella*/EIEC positive.

A QA/QC validation program was administered to each participating laboratory on an annual basis to verify molecular testing capabilities. In brief, the NHRC generated blinded specimens and coordinated with each site laboratory regarding their proficiency testing. The site laboratory identified the blinded sample etiology and reported back to the reference laboratory.

### Statistical methods.

We limited the primary analysis to participants (*n* = 410) for which all variables of interest, including complete testing results for all pathogens, were available. A supplementary analysis (SA) was conducted, examining archived, retrospectively tested samples (*n* = 87) for pathogen data only (metadata were unavailable for this group). All archived specimens were collected from participants who were enrolled in Kenya between January 2013 and December 2015, were male, originated from Europe, and were service members of a non–U.S. military. The only inclusion criterion for this group was that stool grade was 3 or higher and that complete testing results for all pathogens were available. Descriptive statistics were performed, as well as a comparison of single pathogen–, multiple pathogen–, and no pathogen–detected results.

Analyses were performed using SAS software, version 9.4 (SAS Institute, Cary, NC). This study was independently reviewed and approved by the institutional review boards of each participating laboratory.

## RESULTS

From June 2012 to May 2018, stool samples from 410 participants with all variables of interest were collected (Table 3) from Peru (42%), Nepal (40%), Honduras (11%), Thailand (4%), and Egypt (3%). Average age was 29 (SD: 11) years, with age varying across sites (Kruskal–Wallis *P*-value < 0.0001). The oldest participants, on average, were enrolled in Thailand (average age = 38 years, SD: 15) and the youngest, on average, were enrolled in Peru (average age = 27 years, SD: 11). Of the participants enrolled, 54% were female and 46% were male, with proportion of female and male participants varying across sites (Fisher’s exact test *P*-value < 0.0001). Europe was the most common region of origin, with 53% of participants from this region, followed by North America (40%). Most participants enrolled were students (46%), followed by tourists (26%), U.S. military (13%), and other types of travelers (16%), including government and non-governmental organization (NGO) staff.

**Table 3:** Demographic characteristics among acute diarrhea and acute gastroenteritis cases by geographic region and country

Variable	Asia				South/Central America				Middle East		Total	
	Nepal		Thailand		Honduras		Peru		Egypt			
	*n*	%	*n*	%	*n*	%	*n*	%	*n*	%	*n*	%
Country tested	165	40	16	4	44	11	171	42	14	3	410	100
Average age, years (SD)	30 (12)	–	38 (15)	–	33 (8)	–	27 (11)	–	32 (8)	–	29 (11)	–
*P*-value*	< 0.0001											
Sex												
Female	89	54	3	19	9	20	114	67	6	43	221	54
Male	76	46	13	81	35	80	57	33	8	57	189	46
*P*-value†	< 0.0001											
Region of origin												
East Asia	4	80	0	0	0	0	1	20	0	0	5	1
North America	40	25	3	2	44	27	69	42	7	4	163	40
Europe	102	47	9	4	0	0	98	45	7	3	216	53
Oceania	19	76	4	16	0	0	2	8	0	0	25	6
Middle East	0	0	0	0	0	0	1	100	0	0	1	0
Travel type												
U.S. military	1	2	0	0	44	83	2	4	6	11	53	13
Government (U.S. or non-U.S.)	4	100	0	0	0	0	0	0	0	0	4	1
NGO/aid worker	15	100	0	0	0	0	0	0	0	0	15	4
Tourist	89	85	16	15	0	0	0	0	0	0	105	26
Student	18	10	0	0	0	0	169	90	0	0	187	46
Other‡	38	83	0	0	0	0	0	0	8	17	46	11

* Kruskal–Wallis test.

† Fisher’s exact test.

‡ Travel type “other” consisted mostly of individuals describing themselves as “volunteers” (66%).

Across all sites (Table 4), a single pathogen was detected in 43% of specimens, multiple pathogens were detected in 16% of specimens, and 41% of specimens had no pathogen detected. The highest percentages of multiple-pathogen infections were seen in Asia, with 31% of specimens tested in Thailand and 25% of specimens tested in Nepal revealing multiple-pathogen infections. The highest percentages of no pathogen detections were seen in Latin America, with 54% of specimens in Peru and 52% of specimens in Honduras having no pathogen identified. The most frequently detected pathogen in each country was *NoV* (of these, GII was the most common genogroup detected), with the exception of Egypt, where ETEC was most frequently detected. Only Nepal and Kenya (SA) sites detected combination *NoV* GI and GII infections. Infections with *Campylobacter*, EPEC, and EAEC were most commonly seen in Asia (Nepal and Thailand). Across all sites, very few (*n* = 6) infections with *Salmonella* were detected.

**Table 4:** Pathogen results by country and geographic region and country

Pathogen	Asia-Pacific, *n* (%)		South/Central America, *n* (%)		Middle East, *n* (%)	Total by pathogen, *n* (%)
	Nepal	Thailand	Honduras	Peru	Egypt	
*Norovirus*						
Positive	53 (32)	7 (44)	9 (20)	28 (16)	1 (7)	98 (24)
Genogroup I	18 (34)	2 (29)	2 (22)	3 (11)	0 (0)	–
Genogroup II	28 (53)	5 (71)	7 (78)	25 (89)	1 (100)	–
Genogroups I and II	7 (13)	0 (0)	0 (0)	0 (0)	0 (0)	–
Negative	112 (68)	9 (56)	35 (80)	143 (84)	13 (93)	312 (76)
*Campylobacter jejuni*						
Positive	30 (18)	5 (31)	5 (11)	16 (9)	1 (7)	57 (14)
Negative	135 (82)	11 (69)	39 (89)	155 (91)	13 (93)	353 (86)
*Shigella*–enteroinvasive *E. coli*						
Positive	16 (10)	0 (0)	4 (9)	16 (9)	4 (29)	40 (10)
Negative	149 (90)	16 (100)	40 (91)	155 (91)	10 (71)	370 (90)
*Salmonella*						
Positive	3 (2)	3 (19)	0 (0)	0 (0)	0 (0)	6 (1)
Negative	162 (98)	13 (81)	44 (100)	171 (100)	14 (100)	404 (99)
Enteropathogenic *E. coli*						
Positive	16 (10)	5 (31)	0 (0)	10 (6)	1 (7)	32 (8)
Negative	149 (90)	11 (69)	44 (100)	161 (94)	13 (93)	378 (92)
Shiga toxin–producing *E. coli*						
Positive	2 (1)	0 (0)	0 (0)	1 (1)	0 (0)	3 (1)
Negative	163 (99)	16 (100)	44 (100)	170 (99)	14 (100)	407 (99)
Enteroaggregative *E. coli*						
Positive	18 (11)	1 (6)	2 (5)	5 (3)	1 (7)	27 (7)
Negative	147 (89)	15 (94)	42 (95)	166 (97)	13 (93)	383 (93)
Enterotoxigenic *E. coli*						
Positive	35 (21)	1 (6)	6 (14)	16 (9)	6 (43)	64 (16)
Negative	130 (79)	15 (94)	38 (86)	155 (91)	8 (57)	346 (84)
Pathogen combinations						
Single pathogen	74 (45)	10 (63)	17 (39)	69 (40)	7 (50)	177 (43)
Multiple pathogen	42 (25)	5 (31)	4 (9)	10 (6)	3 (21)	64 (16)
None detected*	49 (30)	1 (6)	23 (52)	92 (54)	4 (29)	169 (41)

*E. coli* = *Escherichia coli*.

* Limited to observations with all pathogen reports of “0”; “missing,” or “pending” observations were excluded.

**Figure 3.**
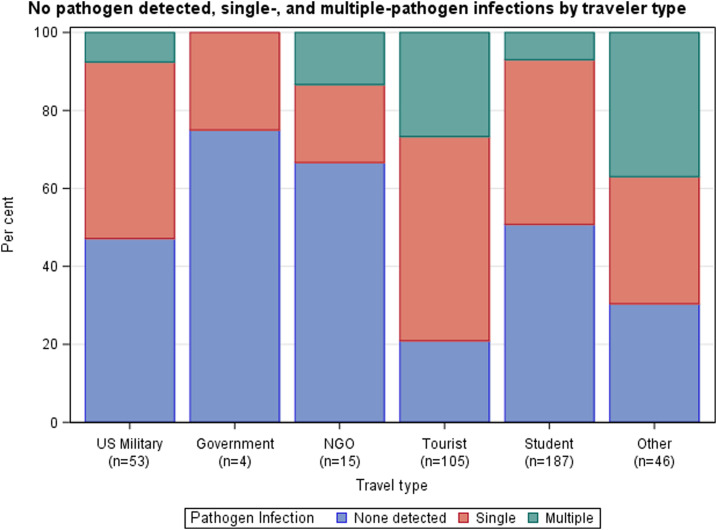
No pathogen detected, single-, and multiple-pathogen infections by traveler type.

**Figure 4.**
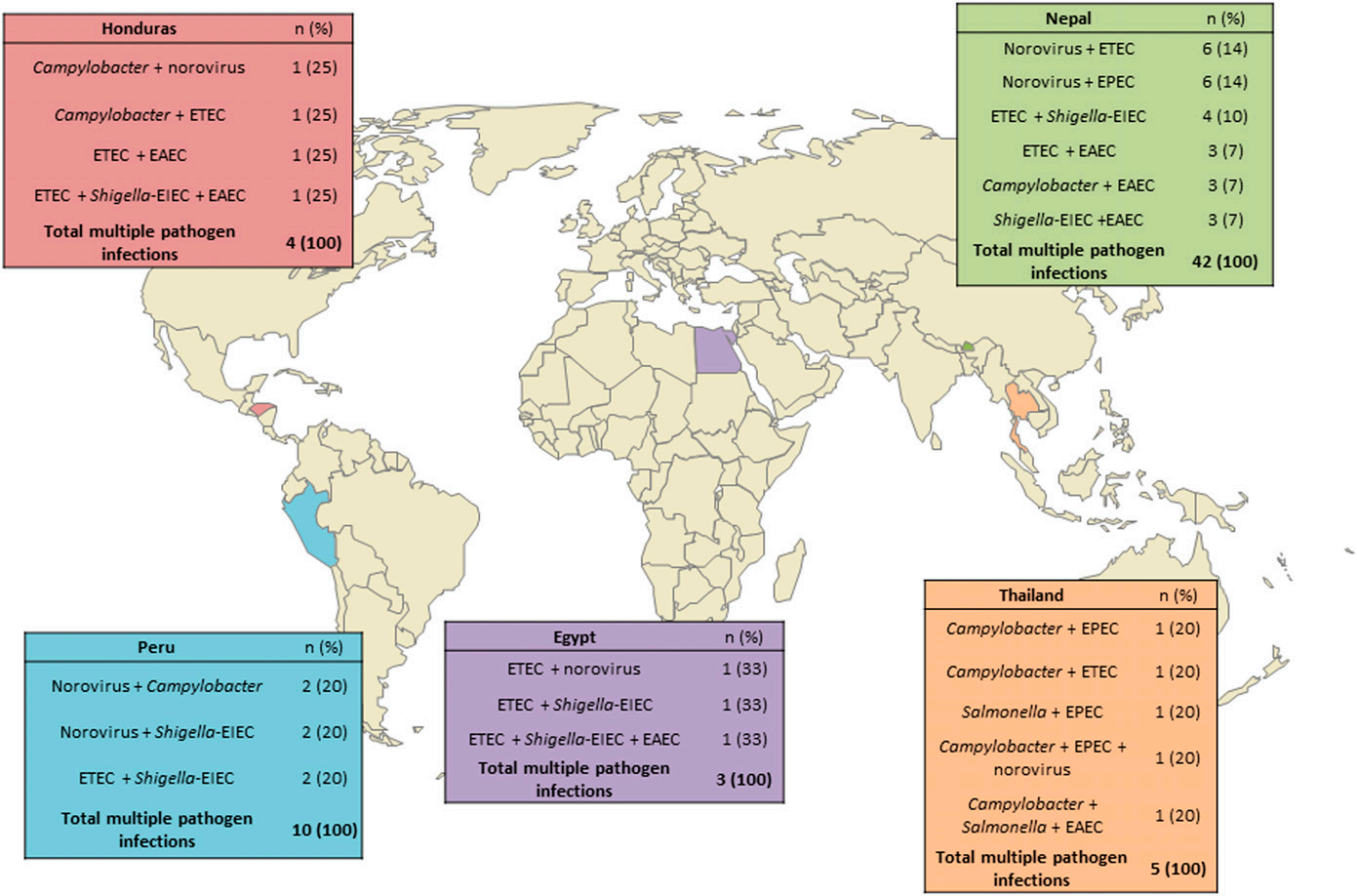
Most frequent pathogen combinations out of multiple pathogen infections by site (not all multiple pathogen infections are listed for each country).

Distribution of single-, multiple-, and no pathogen detections varied by traveler type (Fisher’s exact test, *P* < 0.0001) (Figure 3), with “other” travelers demonstrating the highest percentage of multiple-pathogen infections (17/46, 37%). Most travelers classified as “other” were enrolled in Nepal (38/46, 83%) and reported to be volunteers (25/38, 66%). Tourists were the traveler group with the second highest percentage of multiple-pathogen infections (28/105, 27%). Government travelers, NGO travelers, and students demonstrated the highest percentages of no pathogen–detected results (3/4, 75%; 10/15, 67%; and 95/187, 51%; respectively). The most common multiple-pathogen combinations overall were *NoV*/ETEC (8/62, 13%), *NoV*/EPEC (7/64, 11%), and ETEC/*Shigella*–EIEC (7/64, 11%), with the *NoV*/ETEC and *NoV*/EPEC combinations reported largely among tourists (4/8, 50%; and 6/7, 86%; respectively). Combinations of pathogens within multiple-pathogen infections differed by country site (Figure 4), with *NoV* and DEC combinations seen most frequently in Nepal and *Campylobacter* and DEC combinations seen most frequently in Thailand.

### Supplementary analysis (retrospectively collected samples from Kenya, limited to pathogen data only).

A total of 87 archived specimens with stool grade data meeting inclusion criteria (grades 3–5) were assessed. Enterotoxigenic *E. coli* (29%), followed by *NoV* (17%) and EAEC (15%), were the most common pathogens detected overall among archived specimens (Supplemental Table 1). Among this group, a single pathogen was detected in 39%, multiple pathogens were detected in 17%, and no pathogen was detected in 44% of the archived specimens tested. The most common multiple-pathogen combination was ETEC and EAEC (6/15, 40%)—all other multiple-pathogen combinations in this data subset were observed only once or twice.

## DISCUSSION

This study has examined TD pathogen distribution; single-, multiple-, and no pathogen detected trends; and traveler types across a number of global surveillance sites. Although in the past, there have been multisite studies of children implementing standardized laboratory methods and study designs,^13–15^ to our knowledge, this is the first multisite observational TD study with standardized molecular laboratory methods examining both military and civilian adult travelers.

Although many of our pathogen findings agreed with prior studies, there were notable differences. The most commonly detected pathogen in the main analysis was *NoV*, rather than bacterial pathogens such as ETEC or *Campylobacter* that have been more commonly associated with TD in previous studies,^9,16,17^ although it is possible that *NoV* has been underreported in the past because of short clinical duration, diagnostic methods, or case definitions.^4^ Although *NoV* has long been known to impact military personnel in operational environments because of crowded living situations and lack of development of widespread natural immunity,^18,19^ it has not been considered a leading cause of TD, relative to diarrheagenic bacterial pathogens.^20^ In addition, certain types of travel, such as backpacking, have been found to be a greater risk of *NoV* infection than other travel types,^21^ and our findings of the highest percentages of *NoV* infections in Thailand (44% positive) and Nepal (32% positive) support this, as 100% and 54% of travelers to Thailand and Nepal, respectively, were tourists, and both of these nations are well-known backpacking destinations. This finding highlights the importance of *NoV* as an etiology of TD and underscores the importance of continued vaccine development to prevent illness caused by this significant pathogen.^20^ Our findings of *NoV* GII being the most prominent genotype agree with the findings of others.^22,23^

Enterotoxigenic *E. coli* was the second most frequently detected pathogen overall, and *Campylobacter* was also commonly detected, especially in Thailand and Nepal, in agreement with prior studies.^24,25^ There were few *Salmonella* detections (1% of specimens in the main analysis; 0–19% by region; 0% of specimens from Kenya in the SA), and whereas *Salmonella* was the pathogen implicated for the highest incidence of foodborne infections from 10 sites in the United States (2006–2013),^26^ studies focusing on TD in civilians and deployed/overseas service members have shown *Salmonella* to be detected less frequently relative to other diarrheagenic pathogens.^27^ Our findings of no pathogen detected in 41% of samples agree with previous studies using stool samples collected during acute illness.^4^ Regional differences of pathogen recovery reflect previous work as well,^24,28^ with the highest pathogen recovery found in Southeast Asia and the next highest pathogen recovery found in South Asia. For locations with high numbers of no pathogen detections, the lack of detection could indicate that some etiologies are not being tested for, such as emerging pathogens or toxins that could play a meaningful role in clinical manifestations of TD.^25^

Multiple-pathogen infections were not uncommon (16%), especially among travelers enrolled in the Asian countries participating in our study. This may be attributable, in part, to differences in the traveler types enrolled in Thailand and Nepal versus other sites. Although from a different region, previous work among young Kenyan children has shown that exposure to multiple public locations increased probability of ingesting multiple pathogens.^29^ Because children, like travelers, are naive to enteric pathogens and may be considered a proxy for how adult travelers could respond to pathogen exposure in high-risk areas, this finding may provide insights into the distribution of multiple-pathogen infections among GTD travelers. In Thailand and Nepal, the most frequently enrolled travel populations were tourists and individuals categorized as “other” (the majority of whom [66%] described themselves as “volunteers”). These individuals may have been more likely to visit a larger variety of locations than other traveler categories such as military or student travelers, and such increased exposure to multiple locations might have increased their risk of multiple-pathogen infections.

The pattern of multiple-pathogen infections by both region and traveler type differed from previous work. In their study, Lääveri et al.^30^ tested for all bacterial pathogens that were included in the GTD panel and found that among Finnish travelers experiencing TD, 32% of travelers to Southeast Asia, 60% of travelers to South Asia, 29% of travelers to Latin America, and 52% of travelers to East Africa had multiple pathogen infections. Although our study found a similar pattern of multiple-pathogen infections in Southeast Asia, our results from other geographic locations differed. We found that 25% of participants in Nepal, 9% and 6% (in Honduras and Peru, respectively) of participants in Latin America, and 17% of samples from Kenya (SA) had multiple pathogen infections. The differences between the findings of Lääveri et al.^30^ and our results might have been due to small sample sizes with stratification by region, differences in traveler type and country of origin (Finnish travelers versus any traveler from an OECD member country), or other differences in laboratory protocols.

Nepal, in general, exhibited the greatest variety in pathogens detected, as it was the only site that had positive test results for all pathogens of interest. Nepal may be a riskier area, in general, for TD, as previous work has shown that travel to Nepal has a higher association with TD than other countries, both regionally and globally.^31,32^ It has also been found that studies of TD in U.S. military populations had higher pathogen detection than those conducted in nonmilitary individuals,^28^ although we did not find this in our study. Although reasons for this are not completely clear, the comparatively lower proportions of U.S. military with pathogen detections (53%) in our study might have been related to the high percentage (83%) of U.S. military who were enrolled in Honduras, the country with the highest percentage of no pathogen detections. Of note, when examining only the nine U.S. military members who were not enrolled in Honduras, 78% of these participants had at least one pathogen detected.

There were differences in most frequently detected pathogens among the retrospectively tested samples from Kenya compared to the main analysis. Whereas *NoV* (24%), followed by ETEC (15%), were the most commonly identified pathogens in the primary analysis, ETEC (29%), followed by *NoV* (17%), were the most common pathogens detected overall in the SA. This is in agreement with previous work examining British soldiers in Kenya, revealing ETEC as the most frequently detected pathogen.^27^ Furthermore, EAEC was detected among 15% of samples from the SA, yet only detected in 6% of samples in the main analysis. This higher percentage of EAEC found in the African region differs from Shah et al.,^17^ who found EAEC to be infrequently detected in Africa (3/165, 2%), but is in agreement with later findings of ETEC, EPEC, and EAEC frequently detected among Western military personnel in South Sudan, and *NoV* detected less frequently.^33^ Despite these differences in most common pathogens detected, the distribution of single-, multiple-, and no pathogen–detected results showed similar patterns when comparing the SA with the primary analysis, although these differed from the work of Biswas et al.^33^ who found that nearly 80% of those enrolled in their study had two or more pathogens detected. However, this group used the BioFire Film Array GI panel, which included a wider scope of pathogens than the GTD study, and so may have contributed to the higher proportion of reported coinfections. The most commonly detected multiple pathogen infections via the Film Array GI panel included ETEC, EPEC, and EAEC, all of which were pathogens tested for in the GTD study. Even so, the high sensitivity of the BioFire Film Array is well known, in particular, the potential for false-positive ETEC detections due to cross-reactivity has been described, and therefore, it is unsurprising that many more multiple-pathogen infections were detected in participants with samples tested by the Film Array GI panel alone^34^ than the parallel molecular and culture laboratory approach of the GTD study.

Our study had several limitations that should be considered. Although molecular laboratory methods were harmonized across sites, culture was not standardized and was performed at differing points in individual laboratory workflows. Even so, the impact on our results is likely negligible, as few pathogens would be expected to be detected by culture and not by standardized PCR testing. There were also differences in sample sizes and demographic composition of site participants. Most travelers in our study were enrolled in Nepal (40%) or Peru (42%), and traveler types enrolled at a given site largely depended on the accessibility of these groups to each partnering laboratory. For instance, those enrolled in Nepal tended to be tourists or “other” travelers and were seeking treatment at one of two travel clinics known to provide care for trekkers on adventure travel. Those enrolled in Peru tended to be students and were seeking care at a clinic associated with a Spanish language school in Cusco. There were differences in participant sex distribution by site, and this might have resulted in variation in care-seeking behavior. Previous work has found that women with TD are more likely than men to seek medical care,^9,35^ although the incidence of TD has been reported to be the same between women and men.^36,37^ In addition, data collection was independently carried out by each participating site, and no standardized questionnaire verbiage, formatting, or training was provided to sites. This might have resulted in misclassification bias if there were differences in how sites collected metadata. Considering limitations presented by sample sizes and differing exposures by location is important in interpretation of these findings and in planning for future surveillance efforts.

## CONCLUSION

Harmonization of methods across unique geographic locations is critical for ensuring consistent identification and characterization of enteric pathogens across the DoD laboratory network, and this ultimately results in more comparable data for global assessments, preventive measures, and treatment recommendations.

Future research should examine in greater detail the role of *NoV* in AD and AGE affecting military or civilian travelers; the distribution of single-, multiple-, and no pathogen detected reports among TD cases; and the impact of the most common pathogen combinations on incidence and severity of TD. Exploring these trends according to traveler type may also provide relevant host factor and exposure information that can better explain trends in both multiple-pathogen detection and disease severity. Assessment and evaluation of the variety of factors potentially contributing to TD, including both host and environmental exposure factors, can inform FHP decision-making for military personnel traveling to high-risk areas as well as shape and prioritize future global surveillance and vaccine development activities.

## Supplemental table

Supplemental materials
