# Supplementary material for: A Multisite Network Assessment of the Epidemiology and Etiology of Acquired Diarrhea among U.S. Military and Western Travelers (Global Travelers’ Diarrhea Study): A Principal Role of Norovirus among Travelers with Gastrointestinal Illness
Source: Am J Trop Med Hyg. 2020 Sep 21;103(5):1855–63. doi: 10.4269/ajtmh.20-0053 (PMC7646805; doi:10.4269/ajtmh.20-0053)
Supplement: Supplementary file 1 [file tpmd200053.SD1.docx]

**The following are supplemental files and will be published online only**

| **Supplemental Table. Pathogen identification from retrospectively collected stool specimens, Kenya, January 2013-December 2015.** | |
| --- | --- |
|  |  |
| **Pathogen** |  |
|  |  |
|  | **n (%)** |
| **Norovirus** |  |
| Positive | 15 (17) |
| Genogroup I | 4 (25) |
| Genogroup II | 8 (53) |
| Genogroup I,II | 3 (20) |
| Negative | 72 (83) |
| ***Campylobacter jejuni*** |  |
| Positive | 1 (1) |
| Negative | 86 (99) |
| ***Shigella*-enteroinvasive *E. coli*** |  |
| Positive | 5 (6) |
| Negative | 82 (94) |
| ***Salmonella*** |  |
| Positive | 0 (0) |
| Negative | 87 (100) |
| **Enteropathogenic *E. coli*** |  |
| Positive | 4 (5) |
| Negative | 83 (95) |
| **Shiga toxin-producing *E. coli*** |  |
| Positive | 3 (3) |
| Negative | 84 (97) |
| **Enteroaggregative *E. coli*** |  |
| Positive | 13 (15) |
| Negative | 74 (85) |
| **Enterotoxigenic *E. coli*** |  |
| Positive | 25 (29) |
| Negative | 62 (71) |
| **Pathogen combinations** |  |
| Single pathogen | 34 (39) |
| Multiple pathogen | 15 (17) |
| None detected | 38 (44) |
| **Total** | **87 (100)** |
|  | |
